# Factors Affecting the Microstructure, Tensile Properties and Corrosion Resistance of AA7075 Forgings

**DOI:** 10.3390/ma14195776

**Published:** 2021-10-02

**Authors:** Teng-Shih Shih, Ho-Tieh Hsu, Lih-Ren Hwang

**Affiliations:** 1 Department of Mechanical Engineering, National Central University, Jhongli District, Taoyuan 32001, Taiwan; forgetpastjustbemyself@gmail.com; 2 Department of Mechanical Engineering, Chung Chou University of Science and Technology, Yuanlin 51003, Taiwan; lrhwang@dragon.ccut.edu.tw

**Keywords:** cold and hot forging, misorientation angle grain boundary, tensile properties, corrosion resistance

## Abstract

AA7075 alloys are high strength alloys and are used as an important material for making engineering parts. Forged AA7075 alloys showed significantly decreased toughness when the material was hot deformed at a high temperature. This study investigated the effects of forging parameters on the tensile properties and the microstructure of AA7075 forgings. The tensile properties and corrosion resistance of different forgings were determined to be correlated with their microstructures. The experiment annealed and hot-deformed sample bars at 633 K, cold-deformed them at room temperature (RF), and at sub-zero temperatures (CF). After T73 heat treatment, the microstructures depended on the deformation temperature. This varied significantly, from elongated grains for hot-forged samples to equiaxial grains for cold-deformed samples. The hot-deformed samples had a tensile strength of 592 MPa for UTS, 538 MPa for YS, and 13.4% for elongation. These were stronger but less elongated than the cold-deformed samples. All hot-deformed (HF), RF, and CF samples exhibited mechanical properties that exceeded UTS > 505 MPa, YS > 435 MPa, and an elongation > 13%, and showed moderate corrosion resistance if samples were in contact with a 3.5 wt.% NaCl solution. The toughness of the forgings could be significantly improved by decreasing the forging temperatures. The corrosion resistance of AA7075-T73 forgings was affected by the total grain boundary (GB) lengths per unit area and the 2nd phase particle counts per unit area. Increasing the high-angle grain boundary lengths (HAGBs) per unit area accelerated corrosion and increased the Icorr value.

## 1. Introduction

High-strength aluminum alloys, such as AA7075 alloys, are eminently suited for use in lightweight vehicles, aircraft, and sport items. These alloys contain large amounts of zinc, magnesium, and copper, which form complex intermetallic compounds within the matrix. The precipitates and/or intermetallic compounds acquire a specific morphology due to the application of a heat treatment or from the manufacturing process. T73 heat treatment produces samples with an over-aged condition in order to reduce stress corrosion [1,2]. Intermetallic compounds and fine precipitates in the aluminum matrix significantly affect the mechanical properties and anodic performance of AA7075 alloys [3,4,5,6].

Forging uses a closed die and high power to produce near net-shaped parts, which decreases manufacturing costs [7]. Forging also increases the fracture toughness of 7050 alloys if there is a proper process control [8]. Therefore, AA7075-T73 forgings are suited for making parts for the car industry. However, forging or extrusion can produce a non-uniform macrostructure in the substrate of samples [9]. The structure is significantly affected by the degree of plastic deformation, working temperature, and strain rate. Forged AA7075 alloys show decreased toughness after the material is hot deformed at a high temperature. This study was initiated due to an engineering issue: a thin-walled hub cracked after service. This part was hot forged at a high temperature of 693 K using a close die. This issue was solved by lowering the forging temperature to 633 K. These topics are of interest to understand the change in microstructures and tensile properties when forging temperatures are decreased.

Aluminum alloys that deform at cryogenic temperatures are reported to suppress the dynamic recovery that occurs during plastic deformation, so fine grains feature high-angle grain boundaries [10,11]. One study showed that interaction between high-density dislocations facilitates precipitation, so a high density of nano-sized precipitates is produced [12]. Nano-grains that are less than 500 nm in diameter are present in AA7075 samples subjected to compression forging at cryogenic temperatures [13]. The typical structure of cryogenically deformed 7075-T6 or 7075-T73 alloys exhibits fine grains and fine precipitates.

The mechanical properties of cryo-rolled 7075 alloy include a higher Vickers hardness, UTS, and yield strength for samples that are cold-rolled at room temperature (RT). The tensile properties of the deformed sample are improved by the deformation of ultra-fine grains. There is a higher Vickers hardness, UTS, and yield strength for samples that are cryo-rolled compared to those that are cold-rolled at RT. The tensile properties of the deformed samples are improved by the formation of ultra-fine grains [14].

Sun and coworkers et al. hot-deformed 7075 alloys at more than 623 K and showed that dynamic recrystallization occurs in local areas [15]. The grain size in areas where there is dynamic recrystallization increases as temperature increases and decreases as the strain rate increases [16,17,18]. This study hot-deforms 7075 alloy at a lower temperature of 633 K and a high strain rate (1–10 s^−1^) to increase the tensile properties of 7075 alloy forgings. 

Rokni et al. demonstrated that the peak stress for hot deformed 7075 alloy increases as the deformation temperature decreases and as the strain rate increases [19]. The forging temperature (633 K) was more than 573 K, above which there is a significant increase in the solid solution of MgZn_2_ in the Al matrix [20].

Gupta et al. studied the hot-deformation of AA7075 alloys and showed that compression testing using a high strain rate of 10 s^−1^ results in a deformation band. EBSD tests showed that 12–13% of low angle grain boundaries were detected in the samples [21]. T74 (over-aging) heat treatment causes the grains size of extruded samples to grow and decreases the HAGBs. For a high strain rate and hot compression, grains are mainly low-angle grain boundary lengths (LAGBs) in the matrix [22].

Recently, Shih et al. recorded the change in the microstructure of hot- and cold-forged AA6082 alloy. Dynamic recrystallization (DRX) occurred in the hot-deformed samples. After T6 heat treatment, hot deformed samples have elongated grains with a high aspect ratio. This results in a high tensile strength, but less elongation. Forging at a low temperature or room temperature increases elongation by 16% and toughness by 10% [23].

## 2. Materials and Methods

Extruded AA7075 bars were used for this study. The bars were 55 mm in diameter and were sliced to produce bar samples with a length of 100 mm. Table 1 shows the chemical composition of AA7075 in wt.%. 

After annealing at 513 K for 900 s and 688 K for 2 h, the bar samples were divided into four groups. One group was cooled to 195 K, stored for 600 s in dry ice and then forged using a set of open dies to reduce the thickness by 50%. This set of samples was denoted as CF50. The final forging temperature for the CF50 samples was about 293–313 K. The second group of bar samples were forged at room temperature (RT) using a set of open dies to reduce the thickness by 50%. These samples were denoted as RF50. The final forging temperature for RF50 samples was about 313–333 K. The third and fourth groups of bar samples were heated to 633 K and forged using a set of closed dies to reduce the thicknesses by 50% and 62%, respectively. These were denoted as HF50 and HF62. The final forging temperature for HF50 and HF62 samples was about 503–523 K. These temperatures were lower than the 753 K soaking temperature that was used for the extruded 7075 sample. After soaking for 5–25 min, the samples featured elongated grains [24]. Therefore, the effect of the final forging temperature on grain morphology was not considered in this study.

**Figure 1:**
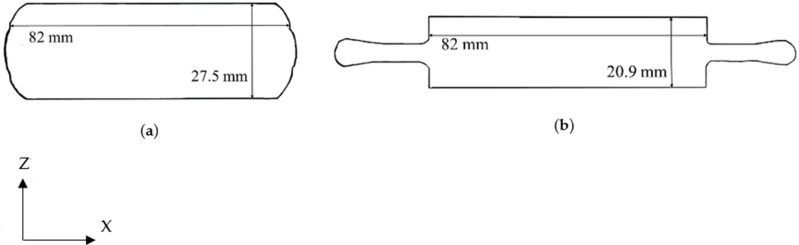
Drawings of (**a**) CF50, RF50, and HF50 (27.5 mm × 82 mm × 105 mm in length), and (**b**) HF62 (20.9 mm × 82 mm × 105 mm in length).

The RF50, CF50 and HF50 samples were subjected to the same degree of deformation. Figure 1a shows their dimensions after forging and Figure 1b shows the dimensions of forged HF62 samples. The Y-axis represents the extrusion direction for the bar samples.

**Figure 2:**
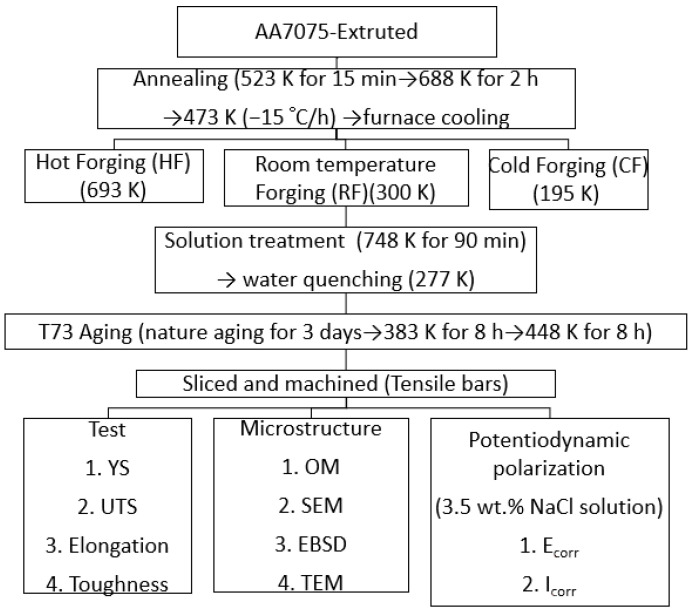
A flow chart used for explaining the experiment and tests.

Figure 2 shows a flow chart describing the experimental process and the conditions used in this study. One forged block was used to manufacture four tensile bar samples with a gauge diameter of 9 mm according to ASTM E8/E8M. The tensile specimens were polished using abrasive paper to achieve a surface roughness of less than (Ra) 0.5 μm. The tensile tests were carried out using an Instron-8801 machine with a strain rate of 0.00042 s^−1^. Details for preparing samples after the tensile tests for optical micrography (OM), energy-dispersive X-ray spectroscopy (EDS) and transmission electron microscopy (TEM) refer to Reference [23]. The microstructure was observed in the Y-Z plane for the forged blocks. The Y and Z axes refer to Figure 1. Potentiodynamic polarization tests were conducted on different samples in a 3.5 wt.% NaCl solution, and the results were recorded by using an Autolab PGSTAT30 Potentiostat (Artisan Technology Group, Champaign, IL, USA). The conditions that were used for running the potentiodynamic polarization test are described in Reference [23].

## 3. Results and Discussion

**Figure 3:**
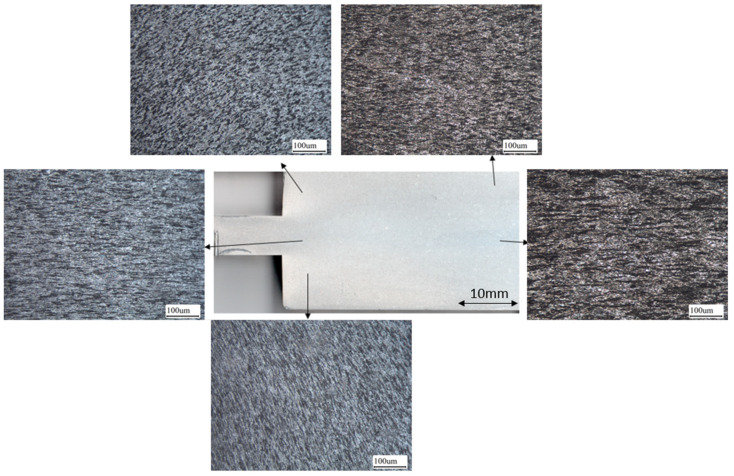
The macrostructures of the as-forged HF62 samples, showing the distribution of flow lines and deformation bands.

Figure 3 shows the macrostructure of the as-forged HF62 sample. Forging flow lines were not uniformly distributed and were tangled at the corners of the forged blocks. These parts were excluded from this study. Four tensile bars were manufactured with an equal distance along the central line of the forging blocks. Effective strain of the forged blocks was calculated according to previous work and varied from 0.3 to 1.35 for HF50 and from 1.05 to 1.4 for HF62 [23].

The mean values and standard deviations of the tested tensile strengths are listed in Table 2. Hot-deformed samples (HF50 and HF62) have greater UTS and YS values than cold-deformed samples (CF50 and RF50). The HF62 sample has extremely large values of 592 MPa for UTS and of 538 MPa for YS, and exhibits moderate elongation (13.4%). The cold-deformed samples (CF50 and RF50) have values of 525 MPa for UTS and 465–467 MPa for YS and exhibit a considerable elongation of 16.4–17.4%. The cold-deformed samples have a higher toughness (>70 × 10^−6^ J/m^3^) and resist tensile fracture better than the hot deformed samples (54–60 × 10^−6^ J/m^3^). The samples differ significantly in terms of tensile strength, and UTS varies from 36.9 MPa for UTS for HF50 to 13.9 MPa for HF62. The tensile properties are affected by the microstructure and the effective strain of the forgings. The HF50 forged block undergoes a wide range of effective strains (0.3 to 1.35) so the deviation is greater than that of the HF62 forged bar, which has values of 1.05 to 1.4. All the forged samples have lower UTS and YS, but are more elongated than samples that are prepared directly from extruded bars (591 MPa for UTS, 564 MPa for YS, and 13.4% in elongation). The forged samples have tensile properties that exceed those of the standard of the Metal Handbook (505 MPa for UTS, 435 MPa for YS, and 13% elongation). 

**Figure 4:**
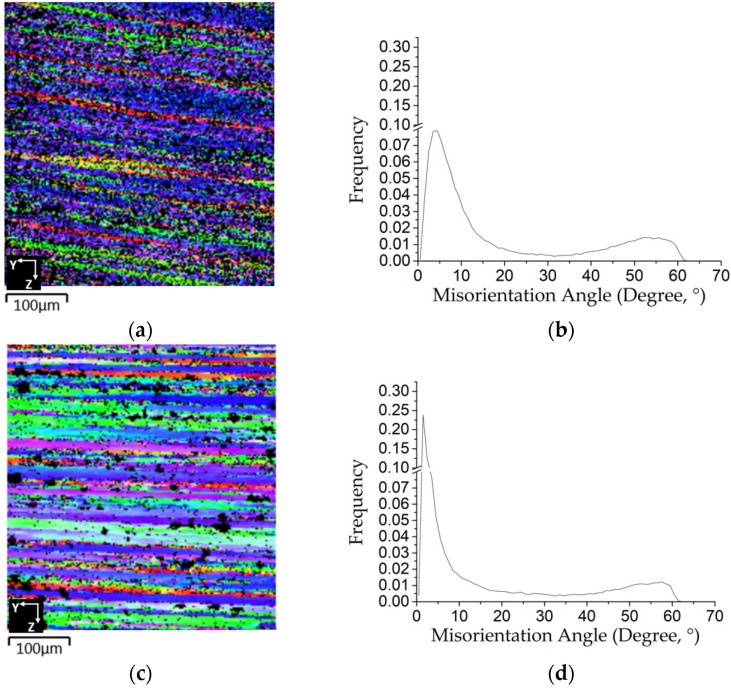
EBSD analysis results: (**a**,**c**) inverse pole figure (IPF); (**b**,**d**) misorientation angle distribution for (**a**,**b**) RF50, (**c**,**d**) HF50.

The EBSD tests gave information about the distribution of the misorientation angle grain boundaries (MAGBs) over the matrix of the forged samples. The results are shown in Figure 4a–d for the as-forged RF50 and HF50. The samples exhibit elongated grains with mainly LAGB values. Figure 4a,b, respectively, show the results for RF50 and Figure 4c,d shows the results for the HF50 samples. The as-forged RF50 has some fine grains that feature HAGB values. Forging at a high compression loading produces a large plastic deformation in the matrix. The HF50 or RF50 bar samples experienced an effective strain of more than 1.34 during forging [23]. AA7075 alloys also contain high amounts of Zn and Mg alloys, so the stacking fault energy and dislocation movement change. Dynamic recrystallization (DRX) and dynamic recovery depend on the strain rate, the degree of deformation, and the working temperature during deformation.

For this study, dynamic recovery is partially suppressed for samples that are forged at room temperature. DRX occurs so there are nano-sized grains in the as-forged RF50, as shown in Figure 4b. Dynamic recovery was seen in the hot forged sample because of the effect of heat and the MAGB values are mostly less than 10°, as shown in Figure 4c,d for the as-forged HF50. Some fine grains are distinguishable in Figure 4c, and these are represented by the measured fractions of HAGBs in Figure 4d.

**Figure 5:**
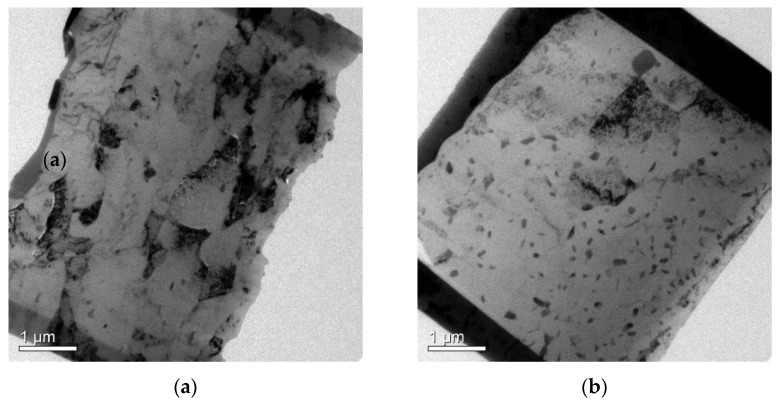
TEM micrograph of (**a**) as-forged RF50 and (**b**) as-forged HF50.

**Figure 6:**
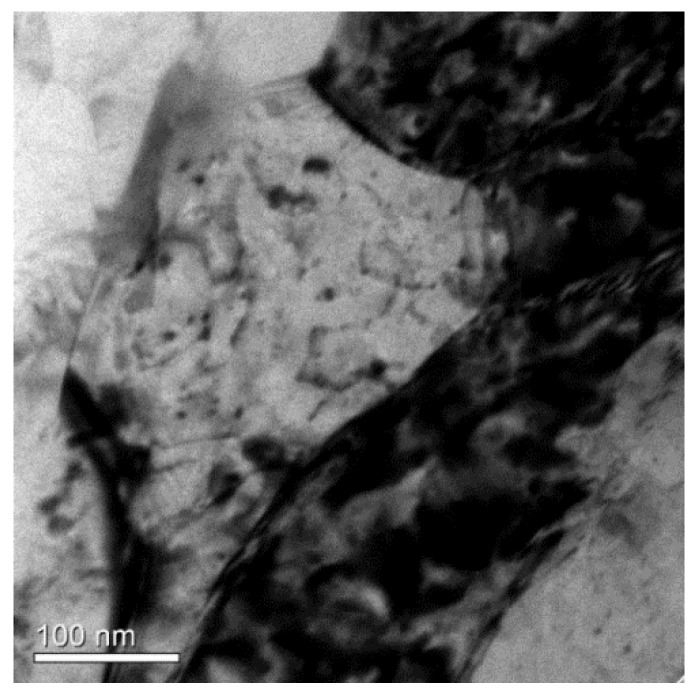
TEM micrograph of as-forged RF50 showing a nano-sized grains with dense dislocation loops.

**Figure 7:**
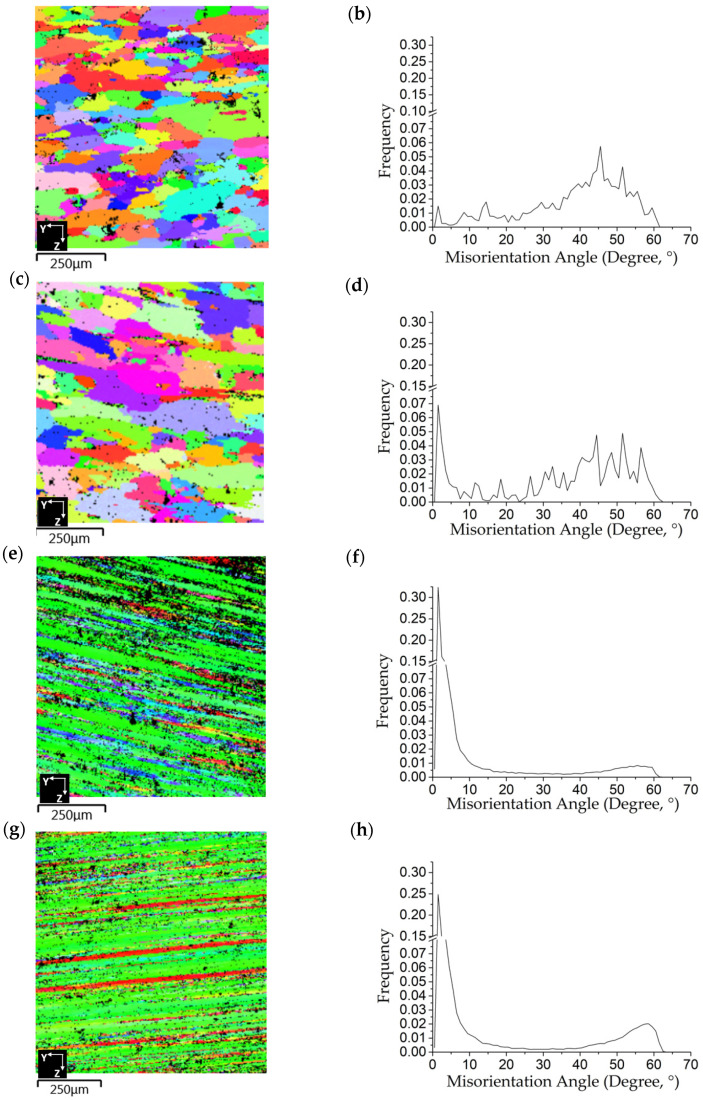
EBSD analysis results: (**a**,**c**,**e**,**g**) inverse pole figure (IPF) and (**b**,**d**,**f**,**h**) misorientation angle distribution for (**a**,**b**) CF50, (**c**,**d**), RF50, (**e**,**f**) HF50, and (**g**,**h**) HF62 after T73 heat treatment.

TEM micrographs for the as-forged RF50 and HF50 show the differences between the two samples. These are, respectively, shown in Figure 5a for RF50 and Figure 5b for HF50. The as-forged RF50 featured more dense dislocation loops in the matrix than the as-forged HF50 sample. There was also a nano-sized grain that was about 300 nm and dense dislocation loops within the grain, as shown in Figure 6, for as-forged RF50. The forged samples exhibited significant changes to the microstructure after T73 heat treatment. Vacancies were present in the cold forged samples due to the low temperatures. These vacancies resulted in the elimination of dislocations in samples during T73 heat treatment. The morphology of CF50 and RF50 grains changed from elongated to equiaxial after heat treatment, as respectively shown in Figure 7a,c. The HAGB values (higher than 15°) are high for the matrix of CF50, as shown in Figure 7b. In the RF50 matrix, there are more fractions of LAGB values. These differences are attributed to the inhibition of dynamic recovery due to a fact that CF50 is treated at a sub-zero temperature. Both HF50 and HF62 featured the elongated grains, as shown in Figure 7e,g. However, DXR was more prevalent in HF62 than in HF50. After T73 treatment, the HF62 had almost twice the number of fine grains with grain boundaries (higher than 45°) as the HF50, as shown in Figure 7f,h. The hot-deformed samples featured elongated grains with mainly LAGB values in the matrix. This observation agrees with the results of Khan and Gupta [21,22]. The cold-deformed samples featured mostly equiaxial grains with HAGB values.

**Figure 8:**
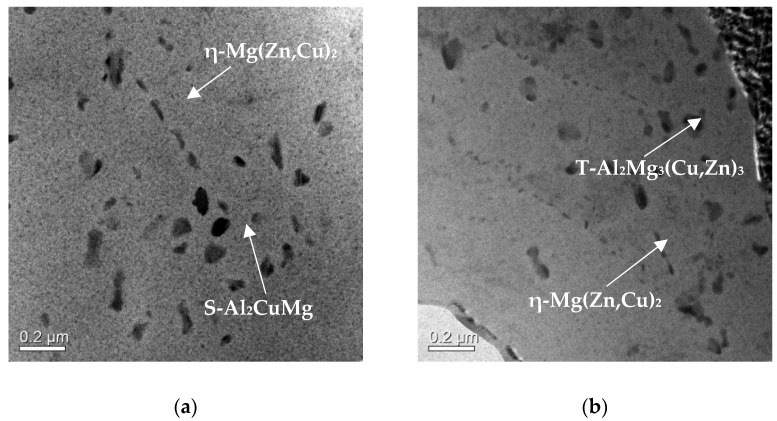
TEM micrograph of different samples: (**a**) RF50 and (**b**) HF50 after T73 heat treatment.

After T73 treatment, dispersoids were produced in the over-aged conditions and these became stable in size. These dispersoids are shown in Figure 8a,b for RF50 and HF50, respectively. The EDX analysis showed the constituted elements of dispersoids, as listed in Table 3. They were Mg(Zn,Cu)_2_, S-Al_2_CuMg and T-Al_2_Mg_3_(Cu,Zn) bases, as described by Hua et al. [25,26]. Angella and co-workers studied the dispersoids in AA7050 alloys and showed that Zn is replaced by Cu to form the Mg(Zn,Cu)_2_ phase [8]. For Al-Mg-Zn-Cu alloys, the MgZn_2_ phase controls the mechanical resistance of the alloys after aging treatment [27].

At an aging temperature of 450 K, the diffusion coefficients for Mg, Zn, and Cu in Al are 1.53 × 10^−19^, 3.97 × 10^−19^ and 1.27 × 10^−20^ m^2^/s, respectively [28]. MgZn_2_ forms initially during aging and then Cu diffuses to increase the size of the precipitated Mg(Zn, Cu)_2_. Grain boundaries fill with point and line defects to accelerate Cu diffusion. The T-Al_2_Mg_3_(Cu, Zn) dispersoids are preferentially located at the grain boundaries [25]. These dispersoids are also detected in the HF50 sample. 

Cabibbo et al. noted that S-Al_2_CuMg dispersoids do not undergo shear deformation if the aluminum alloy is subjected to shear stress [29]. Precipitation hardening is the main strengthening mechanism for the Al-Zn-Mg-Cu series alloys [30]. These dispersoids are abundantly distributed in the matrix of all samples and have sizes of 50–200 nm. Therefore, AA7075 alloys have a greater tensile strength than other series of alloys. The hot-deformed sample features elongated grains with a longer grain boundary length per unit area than the cold-deformed sample. These grain boundaries increase the strength of the matrix for all samples. The hot-deformed samples have an exceptionally high tensile strength, as shown in Table 2.

**Figure 9:**
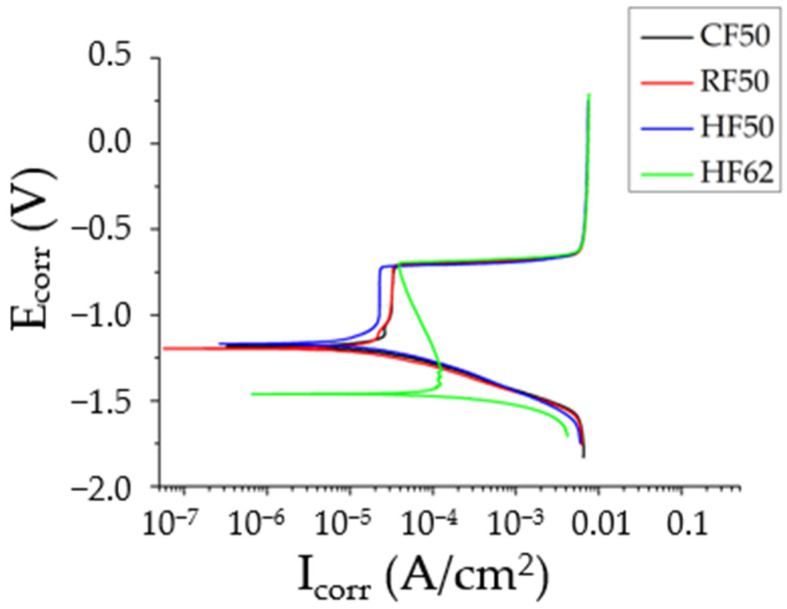
Polarization curves from different samples immersed in a 3.5 wt.% NaCl solution.

**Figure 10:**
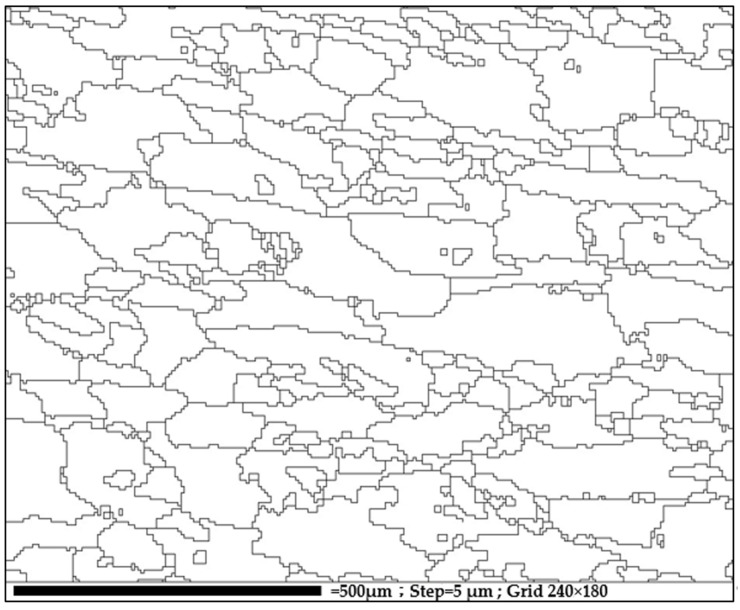
High angle grain boundaries (>15°) measured from RF50.

For the potentiodynamic corrosion test, the polarization curves are shown in Figure 9. The experimental results show that the cold-deformed samples (CF50 and RF50) feature slightly better corrosion resistance (E_corr_: −1.15 to −1.19 V) than the hot-deformed samples (E_corr_: −1.17 to −1.46 V), as shown in Table 4. After T73 treatment, the AA7075 forgings exhibit lower corrosion resistance (E_corr_ and I_corr_ values) than the extruded bar samples (A-T73* in Table 4). The forgings undergo plastic deformation and the grain morphology changes so the microstructure and the corrosion resistance change. The total grain boundary length (GBL) of RF50 was computed from Figure 10, which was derived from EBSD test results in Figure 7c, analyzed using HKL-CHANNEL-5 software. The total GBLs and total 2nd-phase particle counts were the metallurgical factors that affected the E_corr_ values for AA7075 forgings, as shown in Table 4, Table 5 and Table 6.

**Figure 11:**
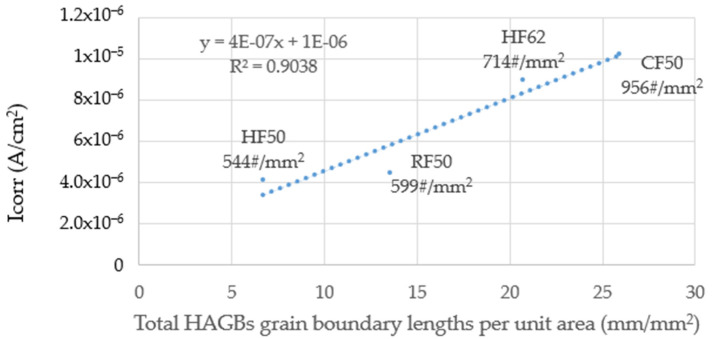
Relationship between I_corr_ and total HAGBs grain boundary lengths per unit area (mm/mm^2^) for different samples; total particle counts (particle size >0.2μm) are included.

Shih et al. noted that HAGBs are attacked by anions, which are seen as gray lines when AA6082-T6 samples are immersed in salt water [23]. The electron current underneath the corroded particles increases, thus, corrosion is accelerated. The total lengths of HAGBs and 2nd phase particle counts per unit area synergistically accelerate corrosion so the I_corr_ value increases [23]. For this study, CF50 has the longest HAGBs length per unit area and the maximum 2nd phase particle counts per unit area (956 counts/mm^2^) so the I_corr_ and I_pit_ values are the highest out of all the samples, as shown in Figure 11 and Table 4. HF50 features the shortest HAGBs per unit area and particles at 544 counts/mm^2^ so it has the lowest I_corr_ value for all samples. The 2nd phase particle counts and the HAGBs lengths were used to determine the I_corr_ values. Table 6 shows the measured 2nd phase particle counts per unit area for different samples. The particle size ranged from 0.2 µm to 10–15 µm.

The 2nd phase particles are believed to contain Mg, Zn, Cu, and some Fe [31,32]. The CF50 obtained the longest HAGBs per unit area in combination of the greatest amounts of particles (956 counts/mm^2^) to accelerate corrosion and had the maximum I_corr_ value. The HF50 and RF50 received similar I_corr_ values but RF50 had longer HAGB lengths per unit area and more particle counts. This could be affected by the locations of the particles at, or near, HAGBs, which is not discussed in this study.

**Figure 12:**
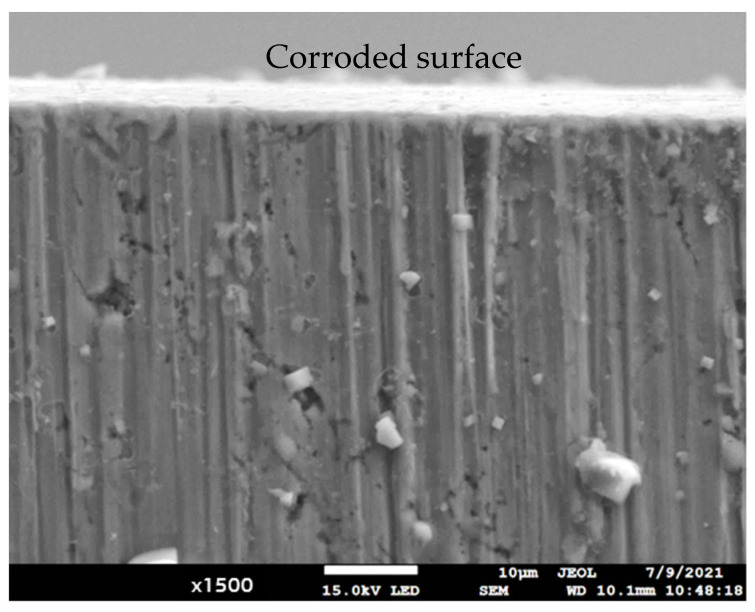
The corroded HF62 sample; including the oxide layer on the surface, trapped fine particles in the substrate and corrosion cracks.

Figure 12 shows the corroded HF62 after the polarization test. An oxide layer was deposited onto the corroded surface. During the test, chloride ions attacked and diffused preferentially into the substrate of the sample via HAGBs. The 2nd phase particles with a size of less than 0.2 μm to about 8 μm were located underneath the sample surface, as shown in Figure 12. If a particle was located at, or near, the HAGBs, it linked the corrosion cracks and likely accelerated corrosion. Both the 2nd phase particles and the HAGBs affect the corrosion resistance of samples. 

## 4. Conclusions

A series of forging parameters were arranged and studied in the experiments. Results indicated that decreasing forging temperature to RT could be effective at enhancing the toughness from 53.9 × 10^−6^ J/m^3^ to 70.5 × 10^−6^ J/m^3^. This is significant to get an increase of about 40% in toughness to resist tensile fracture. These experimental data are valuable for materials designers who plan to apply forged AA7075 alloys in constructing engineering parts. For technical databases, the important results could be summarized as follows; hot forgings: 547–590 MPa in UTS, 492–538 MPa in YS, 13–14% elongation and moderate corrosion resistance of E_corr_ (−1.17 V to −1.46 V) and I_corr_ (4.1 × 10^−6^ to 8.9 × 10^−6^ A/cm^2^); cold forgings enhanced elongation (16–17%) and toughness (71 × 10^−6^ J/m^3^) and resistance to tensile fracture, enhanced E_corr_ values (−1.15 to −1.19 V) and I_corr_ values (1.02 × 10^−5^ to 4.45 × 10^−6^ A/cm^2^).

The corrosion resistance of AA7075 forgings was affected by the resultant microstructures. Increasing HAGBs lengths per unit area and 2nd-phase particle counts accelerated the corrosion to increase the I_corr_ value when the forged AA7075 alloys were in contact with salt water.

## Figures and Tables

**Table 1:** Chemical Composition of AA7075 (wt.%) Bar Samples That Were Used for This Study.

Element	Zn	Mg	Fe	Mn	Cr	Ti	Si	Cu
Content	5.63	2.45	0.12	0.03	0.22	0.03	0.09	1.57

**Table 2:** Measured Tensile Strength (UTS, YS, and total elongation TEL) for Different Samples after T73 Treatment (listed values are the average of four tests).

Sample Code	Ultimate Tensile Stress (MPa)	Yield Stress (MPa)	Total Elongation (%)	Toughness (10^−6^ J/m^3^)
7075 *	505	435	13	NA
7075 **	591 (9.8)	564 (12)	13.4 (0.5)	NA
CF50	525.2 (3.4)	467.0 (10.9)	17.4 (1.7)	71.3 (2.1)
RF50	525.2 (26.8)	465.7 (28.1)	16.4 (2.5)	70.5 (4.1)
HF50	547.4 (36.9)	491.8 (39.7)	14.0 (2.0)	53.9 (3.4)
HF62	591.8 (13.9)	538.3 (11.4)	13.4 (0.9)	60.1 (2.9)

Tel: total elongation; toughness derived from the stress–strain curves of different tensile bars. Deviations were listed in the parentheses. * 7075-T73 standard from Metals Handbook, Vol.2–Properties and Selection: Nonferrous Alloys and Special-Purpose Materials, ASM International 10th Ed. 1990. ** 7075-T73 as-received bar samples.

**Table 3:** EDX Spectrum and Constituted Phases for Dispersoids in Different Samples. (wt.%).

Al	Zn	Mg	Cu	Si	Fe	Mn	Cr	Ti	Phase	Sample
33.06	35.19	9.99	20.32	0.14	0.76	0.14	0.22	0.08	η-base *	CF50
69.98	3.27	6.30	14.39	0	0.52	0.13	5.38	0	S-base **	CF50
57.13	15.52	6.23	18.54	0.01	0.74	0	1.79	0	η-base *	RF50
69.03	3.42	5.57	15.73	0	0.48	0.3	5.39	0.05	S-base **	RF50
25.05	38.19	13.08	21.34	0	0.96	0	1.08	0.27	T-base ***	HF50
56.80	12.77	5.54	22.61	0.19	1.29	0	0.51	0.25	η-base *	HF50
47.89	4.16	8.09	29.23	0	0.92	0.10	9.28	0.29	S-base **	HF62
8.69	47.79	16.55	25.20	0.1	1.3	0.19	0.08	0.06	η-base *	HF62

*, **, *** Reference [25]: Hua et al. *Mater. Des.* **2020**, *196*, 109–192. Reference [26]: Mondal, C.; Mukhopadhyay, A.K. *Mater. Sci. Eng. A.* **2005**, *391*, 367–376.

**Table 4:** Polarization Curves for Different Samples Immersed in a 3.5 wt.% NaCl Solution.

Sample	E_corr_(V)	I_corr_(A/cm^2^)	E_pit_(V)	I_pit_(A/cm^2^)
A-T73 *	−1.11	2.71 × 10^−7^	NA	NA
CF50	−1.15	1.02 × 10^−5^	−0.70	2.30 × 10^−4^
RF50	−1.19	4.45 × 10^−6^	−0.71	3.42 × 10^−5^
HF50	−1.17	4.12 × 10^−6^	−0.72	2.43 × 10^−5^
HF62	−1.46	8.96 × 10^−6^	−0.69	3.89 × 10^−5^

* The AA7075 extruded bar samples after solution treatment (748 K/90 min) and T73 aging (Nat-ural aging 3 days plus 383 K/8h and 448 K/8h).

**Table 5:** Total Grain Boundary Lengths Per Unit Area (mm/mm^2^) for Different Samples.

Sample Code	Total Grain Boundary Lengths per Unit Area (mm/mm^2^)
CF50	28.5
RF50	17.4
HF50	37.2
HF62	66.9

**Table 6:** Measured 2nd Phase Particle Size Distribution for Samples from Optical Micrographs; Particles Counted from 1 µm to Maximum Size in Each Sample (CF50, RF50, HF50, and HF62).

Samples	2nd Phase Particle Counts, (#/mm^2^)					Max. Diameter	Average Diameter
	0.2–1 μm	1–5 μm	5–10 Μm	>10 μm	Total	2nd Phase Particles (μm)	
CF50	663	281	11	1	956	14.4	1.1
RF50	322	269	8	0	599	9.6	1.3
HF50	317	216	11	0	544	9.0	1.2
HF62	570	199	3	0	714	8.9	0.9

## Data Availability

Not applicable.
